# Human eosinophil adhesion and degranulation stimulated with eotaxin and RANTES *in vitro*: Lack of interaction with nitric oxide

**DOI:** 10.1186/1471-2466-8-13

**Published:** 2008-08-12

**Authors:** Letícia Lintomen, Gilberto Franchi, Alexandre Nowill, Antonio Condino-Neto, Gilberto de Nucci, Angelina Zanesco, Edson Antunes

**Affiliations:** 1 Department of Pharmacology, Faculty of Medical Sciences, State University of Campinas (UNICAMP), Campinas (São Paulo), Brazil; 2 Department of Physical Education; Institute of Bioscience, University of Sao Paulo State (UNESP), Rio Claro (SP), Brazil

## Abstract

**Background:**

Airway eosinophilia is considered a central event in the pathogenesis of asthma. The toxic components of eosinophils are thought to be important in inducing bronchial mucosal injury and dysfunction. Previous studies have suggested an interaction between nitric oxide (NO) and chemokines in modulating eosinophil functions, but this is still conflicting. In the present study, we have carried out functional assays (adhesion and degranulation) and flow cytometry analysis of adhesion molecules (VLA-4 and Mac-1 expression) to evaluate the interactions between NO and CC-chemokines (eotaxin and RANTES) in human eosinophils.

**Methods:**

Eosinophils were purified using a percoll gradient followed by immunomagnetic cell separator. Cell adhesion and degranulation were evaluated by measuring eosinophil peroxidase (EPO) activity, whereas expression of Mac-1 and VLA-4 was detected using flow cytometry.

**Results:**

At 4 h incubation, both eotaxin (100 ng/ml) and RANTES (1000 ng/ml) increased by 133% and 131% eosinophil adhesion, respectively. L-NAME alone (but not D-NAME) also increased the eosinophil adhesion, but the co-incubation of L-NAME with eotaxin or RANTES did not further affect the increased adhesion seen with chemokines alone. In addition, L-NAME alone (but not D-NAME) caused a significant cell degranulation, but it did not affect the CC-chemokine-induced cell degranulation. Incubation of eosinophils with eotaxin or RANTES, in absence or presence of L-NAME, did not affect the expression of VLA-4 and Mac-1 on eosinophil surface. Eotaxin and RANTES (100 ng/ml each) also failed to elevate the cyclic GMP levels above baseline in human eosinophils.

**Conclusion:**

Eotaxin and RANTES increase the eosinophil adhesion to fibronectin-coated plates and promote cell degranulation by NO-independent mechanisms. The failure of CC-chemokines to affect VLA-4 and Mac-1 expression suggests that changes in integrin function (avidity or affinity) are rather involved in the enhanced adhesion.

## Background

Airway eosinophilia is considered a central event in the pathogenesis of asthma. The toxic components of eosinophils are thought to be important in inducing bronchial mucosal injury and dysfunction [1]. Evidences suggest that recruitment of eosinophils into sites of inflammation is a multifactorial and multistep process, involving eosinophil-endothelial interactions through adhesion molecules and local generation of chemotactic agents that direct cell migration into the inflamed airways [2]. Thus, adhesion molecules and chemokines play key roles in selective eosinophil accumulation [3]. The integrins macrophage adhesion molecule-1 (Mac-1, CD11b/CD18, αMβ2) and very late antigen-4 (VLA-4, CD49d/CD29, α4β1) are the most important integrins responsible for the firm adhesion of eosinophils to the endothelium through their ligands the intercellular adhesion molecule (ICAM)-1 and the vascular cell adhesion molecule (VCAM)-1 [4]. The integrin VLA-4 is constitutively expressed in eosinophils [5], whereas Mac-1 is expressed in eosinophils stimulated with different class of mediators such as platelet-activating factor (PAF), granulocyte macrophage colony stimulating factor (GM-CSF), and N-formyl-methionyl-leucyl-phenylalanine (fMLP) [6-8]. Both Mac-1 and VLA-4 integrins have a role in the degranulation and superoxide anion production in stimulated eosinophils [6,9], and thus can also be referred as mediators of eosinophil functions. These integrins can also bind to extracellular matrix components such as serum, fibrinogen and fibronectin. Adhesion of eosinophils to fibronectin via VLA-4 [8] prolongs cell survival by Fas antigen signalling, indicating a role for integrin adhesion and signaling in regulating eosinophil function and death [10]. The firm adhesion of eosinophils to fibronectin is mediated through the binding of eosinophil-expressed VLA-4 to the connecting segment 1 (CS-1) region of fibronectin. Eosinophils bind to CS-1 with high avidity, an effect inhibited with neutralizing antibodies to α4 integrins expressed by eosinophils or with neutralizing antibodies to CS-1 [11].

Eotaxin and RANTES (Regulated upon activation normal T cell expressed and secreted) are CC-chemokines responsible for selective eosinophil chemotaxis and transendothelial migration in airways of allergic subjects [12-15]. Selective activation of VLA-4 by eotaxin has provided one mechanism of more efficient recruitment of eosinophils to the lung [16]. In addition, eosinophil priming with interleukin-5 (IL-5) results in a synergistic increase in transendothelial migration in response to RANTES [17] and eotaxin [18]. In fact, interactions of IL-5 and chemokines induce a change in the affinity of VLA-4 in responding leukocytes [5].

Several studies have demonstrated the involvement of nitric oxide (NO) on eosinophil recruitment, but this is still a matter of controversy. Rats treated with the nitric oxide synthase (NOS) inhibitor N^ω^-nitro-L-arginine methyl ester (L-NAME) shows a marked reduction in rat eosinophil migration *in vivo *in an allergic inflammation model [19]. In vitro studies have showed that incubation of rat or human eosinophils with NOS inhibitors attenuate the *in vitro *chemotaxis, suggesting that NO has a role in cell locomotion [20,21]. Previous studies have investigated the functional interactions between NO and CC-chemokines. Young et al. [22] showed that increased number of eosinophils in bronchoalveolar lavage (BAL) fluid of challenged monkeys is markedly increased at 24 h post-challenge, and that is accompanied by higher levels of both exhaled NO and eotaxin in BAL fluid. In rhinitis patients, eotaxin increased the number of eosinophils in the nasal lavage fluid, and that was also accompanied by elevated nasal NO levels [23]. In contrast, in murine models of asthma, selective inhibition of inducible NOS resulted in reduction of the pulmonary eosinophil migration [24,25], which was associated with increased expression of the CC-chemokine monocyte chemoattractant protein-1 (MCP-1) in the lung tissue [26]. Moreover, NO (or NO donors) have also been shown to inhibit the production of RANTES [27]. Nitric oxide via peroxynitrite (ONOO^-^) formation was also shown to reduce the eotaxin-induced eosinophil migration [28]. However, the modulatory role of NO in the CC-chemokines-mediated eosinophil functions is still conflicting. Therefore, the present study was designed to investigate the modulatory effect of NO in the enhanced eosinophil adhesion and degranulation induced by the CC-chemokines eotaxin and RANTES in vitro, and the expression of VLA-4 and Mac-1 in the eosinophil surface.

## Methods

### Human eosinophil isolation

Blood was collected from healthy volunteers who were not under medication. Informed consent and approval from the local ethics committee were obtained before the study. Eosinophils were isolated from peripheral blood as originally described by Hansel *et al*. [29] with minor modifications. Briefly, 60–120 ml of blood collected in 3.13% (w/v) sodium citrate was diluted 1:1 with phosphate buffered saline (PBS) and 35 ml of diluted blood was overlaid onto a 15 ml Percoll gradient (1,088 g/ml, pH 7.4, 340 mosmol/Kg H2O). Gradients were centrifuged at 1000 × *g *for 20 min, 4°C (Hermle model Z360k centrifuge, Germany) and the pellet containing red cells and granulocytes was collected. Red cells were lysed with lysing buffer (155 mM NH4Cl, 10 mM KHCO3 0.1 mM EDTA), and the remaining granulocytes were washed and then incubated with anti-CD16 immunomagnetic microbeads for 30 min before passing on a steel-matrix column in a magnetic field and CD16-negative eosinophils were collected. Eosinophils were resuspended in RPMI-1640 medium (without phenol red), pH 7.2 (> 92% eosinophils, contaminating cells were mononuclear cells).

### Experimental Protocols

After the purification of human isolated eosinophils, the following in vitro assays were carried out: cell viability (MTT assay), adhesion to fibronectin-coated plates, EPO release (cell degranulation), cGMP level measurement and flow cytometry analysis for the adhesion molecules VLA-4 and Mac-1. These assays have been performed in eosinophils stimulated with either eotaxin or RANTES, in the absence and/or in the presence of L-NAME, as detailed below.

### MTT assay

Cell viability was estimated using the tetrazolium salt reduction test (MTT assay) by eosinophils after exposure to chemokines. Isolated eosinophils were resuspended to a concentration of 2 × 10^6 ^cells/ml in RPMI-1640 medium (without phenol red) and then were exposed or not to eotaxin or RANTES (10, 100 and 1000 ng/ml each) for 2, 3 or 4 h at 37°C in a humified atmosphere. Eosinophils (100 μl/well), treated or not, and 3-(4,5-dimethylthiazol-2-yl)-2,5 diphenyl tetrazolium bromide (MTT, 10 μl/well, 5 mg/ml in PBS) were added in triplicate to a 96-well plates. Cells were allowed to incubate for 3 h at 37°C and 5% CO2. After incubation, 100 μl of 10% SDS in 0.01 M HCl was added to each well. Cell samples were then incubated for 18 h at 37°C, 5% CO2 and absorbance measured at 540 nm in a microplate reader (Multiscan MS, Labsystems, USA).

### Eosinophil adhesion assay

96-well plates were prepared by coating individual wells with 60 μl of fibronectin solution (20 μg/ml in PBS) overnight at 4°C. Wells were then washed twice with PBS before blocking non-coated sites with 0.1% (w/v) BSA for 60 min at 37°C. Wells were washed twice again with PBS before allowing plates to dry. Eosinophils (50 μL of 7 × 10^4 ^cells/ml) were incubated with either eotaxin (10–1000 ng/ml) or RANTES (10–1000 ng/ml) for 2, 3 and 4 h in the absence or in the presence of 0.1 mM of L-NAME (or its inactive enantiomer D-NAME) prior to addition to fibronectin-coated wells. Platelet-activating factor (PAF; 10^-6 ^M) was used as a positive control [30]. After 2, 3 or 4 h of incubation, the eosinophils were added in a volume of 50 μl of RPMI-1640 medium without phenol red (7 × 10^4 ^cells/ml) to the coated wells. Cells were allowed to adhere to wells for 30 min at 37°C, 5% CO2. After incubation, non-adhered cells were removed and the remaining cells were washed twice with PBS. Fifty μl of RPMI-1640 medium were added to each well, and varying concentrations of the original cell suspension were added to empty wells to form a standard curve. Eosinophil adhesion was calculated by measuring residual eosinophil peroxidase (EPO) activity of adherent cells [9]. Fifty μl of EPO substrate (1 mM H2O2, 1 mM o-phenylenediamine and 0.1% Triton X-100 in Tris buffer, pH 8.0) were added to each well. After 30 min at room temperature, 25 μl of 4 M H2SO4 were added to each well to stop the reaction and absorbance measured at 490 nm in a microplate reader. Adherence was calculated by comparing absorbance of unknowns to that of the standard curve. In order to exclude the possibility that enhanced eosinophil adhesion to chemokines or L-NAME reflects augmented EPO activity, varying number of eosinophils (20 to 200 μL of 7 × 10^4^cells/ml) were incubated with RPMI alone, eotaxin (100 ng/ml), RANTES (100 ng/ml) or L-NAME (0.1 mM) 4 h in non-coated wells. The EPO measurement in all samples did not differ significantly among each other in any condition used (data not shown), indicating that EPO activity can be used as a marker of eosinophil adhesion. Each experiment was carried out in triplicate (*n *= 6 different individuals).

### Eosinophil degranulation assay

Eosinophil peroxidase release was measured in the supernatants by the o-phenylenediamine (OPD) method [31]. All tubes used were precoated with 2.5% human serum albumin for 2 h at 37°C to block nonspecific activation of eosinophils, and were washed with 1 ml of PBS once before aliquots of eosinophils were added. Ninety μl of eosinophils (5 × 10^6 ^cells/ml in RPMI-1640 medium without phenol red) stimulated or not with eotaxin (100 ng/ml) or RANTES (100 ng/ml), in the absence or in the presence of 0.1 mM of L-NAME (or of 0.1 mM D-NAME), were incubated for 4 h at 37°C, 5% CO2. The calcium ionophore A23187 (5 μM, 30 min) was used as a positive control [32]. At the end of the incubation period the reaction was stopped by cooling on ice for 3 min. The samples were centrifuged at 250 × *g *for 15 min at 4°C and the cells discarded. Aliquots of the supernatants (50 μl; triplicate) were dispensed into each well of a microplate, and 50 μl of eosinophil peroxidase substrate (1 mM H2O2, 1 mM OPD and 0.1% Triton X-100 in Tris buffer, pH 8.0) was added to each well. Measurement of EPO activity was measured at 490 nm using a microplate reader (Multiscan MS, Labsystems, USA), as described above. Each experiment was carried out in triplicate (*n *= 4 different individuals).

### Flow cytometry

Eosinophils were incubated with either eotaxin (100 ng/ml) or RANTES (100 ng/ml) for 4 h in the absence and/or in the presence of L-NAME. Eosinophils were also incubated with PAF (10^-6 ^M) using the same experimental conditions. Expression of adhesion molecules on the surface of eosinophils was detected using flow cytometry. After 2, 3 or 4 h of incubation, eosinophils were washed and incubated (100 μL of 3 × 10^6 ^cells/ml) for 20 min at 4°C with 5 μl of human serum. Cells were washed and incubated for 20 min at 4°C with 5 μl of RPE-conjugated mouse IgG1, k monoclonal immunoglobulin isotype control, RPE-conjugated mouse anti-human CD11b/Mac-1 monoclonal antibody or RPE-conjugated anti-human CD49d monoclonal antibody, after which they were washed and fixed in 0.3 ml 0.5% paraformaldehyde. Cells were analysed on a Becton-Dickinson FACScalibur (San Jose, USA). The mean fluorescence intensity was compared to that of isotype control reacted cells.

### Extraction and measurement of cyclic guanosine-3', 5'-monophosphate (cGMP) from eosinophils

Eosinophils were isolated and resuspended to a concentration of 1 × 10^7 ^cells/ml in PBS. Cells were incubated with the phosphodiesterase inhibitor 3-isobutyl-l-methyl-xanthine (IBMX; 2 mM) for 30 min at room temperature. Then, eosinophils were incubated (37°C, humidified atmosphere) for 30 min or 4 h with or without eotaxin (or RANTES) and/or L-NAME. The NO donor sodium nitroprusside (SNP; 0.1 mM) were used as positive control in the cGMP assays. Next, the reaction was interrupted by the addition of cold acidified absolute ethanol to a final concentration of 67% (v/v), and samples were vigorously agitated by hand for 30 sec. Cell samples were then incubated on ice for 30 min before centrifuging at 4000 *g *for 30 min at 4°C. The supernatants were collected, retained and the precipitates washed with 0.5 ml 67% (v/v) acidified ethanol before centrifuging again at 14,000 *g *for 5 min at room temperature. The supernatants from these washed samples were collected and added to the first supernatants collected and dried at 55–60°C under a stream of nitrogen in a water bath and stored at -20°C until measurement of cGMP. Cyclic GMP in 1.5 × 10^6 ^cells/well was measured using Cayman kit (Ann Arbor, USA). The samples for measurement of cGMP were previously acetylated, following the manufacturer recommendations. Each experiment was carried out in triplicate using 03 healthy volunteers.

### Materials

The system used for eosinophil purification, complete with columns and microbeads, was acquired from Miltenyi Biotec Inc. (Auburn, CA, USA). The antibodies used to flow cytometry assays were purchased from BD Biosciences Pharmingen (RPE-conjugated mouse IgG1, k monoclonal immunoglobulin isotype control, RPE-conjugated mouse antihuman CD11b/Mac-1 monoclonal antibody and RPE-conjugated anti-human CD49d monoclonal antibody; San Jose, USA). Recombinant human eotaxin and RANTES were obtained from R&D Systems (Saint Louis, USA). All other products were purchased from Sigma Chem. Co (St. Louis, MO, USA), unless otherwise stated.

### Statistical analysis

Results are expressed as means ± S.E.M and analysis of variance (ANOVA) for multiple comparisons followed by Tukey's test, or unpaired Student's *t*-test were performed when appropriate. A value of P < 0.05 was taken as significant.

## Results

### Cell viability

The MTT reduction assay was performed to ascertain the eosinophil viability during incubation periods of 2, 3 and 4 h in the presence of either eotaxin or RANTES (10–1000 ng/ml). Our data showed that eosinophils remained viable in all concentrations and incubation periods studied (Table 1). A concentration-dependent increase (P < 0.05) in cell activity was rather detected in most of our experimental conditions.

**Table 1:** MTT reduction assay in stimulated eosinophils in vitro.

**Treatment**	**MTT Reduction (% control)**		
	
	**2 h**	**3 h**	**4 h**
Eotaxin			
(10 ng/ml)	84.6 ± 6.6	113.0 ± 6.1	91.0 ± 2.7
(100 ng/ml)	113.8 ± 5.0*	130.7 ± 2.3*	127.3 ± 3.6*
(1000 ng/ml)	124.7 ± 5.0*	138.4 ± 2.0*	146.0 ± 11.8*
RANTES			
(10 ng/ml)	104.8 ± 1.6	128.8 ± 8.0*	105.8 ± 1.0
(100 ng/ml)	118.9 ± 2.8*	133.4 ± 3.6*	138.4 ± 6.0*
(1000 ng/ml)	117.4 ± 3.8*	118.0 ± 1.0*	147.0 ± 6.9*

Eosinophil suspension (2 × 10^6^cells/ml) was incubated for 2, 3 or 4 h with eotaxin (10–1000 ng/ml) or RANTES (10–1000 ng/ml). Each experiment was carried out in triplicate (*n *= 3). Results are expressed as MTT reduction (% control). *P < 0.05 compared to respective untreated cells.

### Eotaxin and RANTES-induced eosinophil adhesion to fibronectin

**Figure 1:**
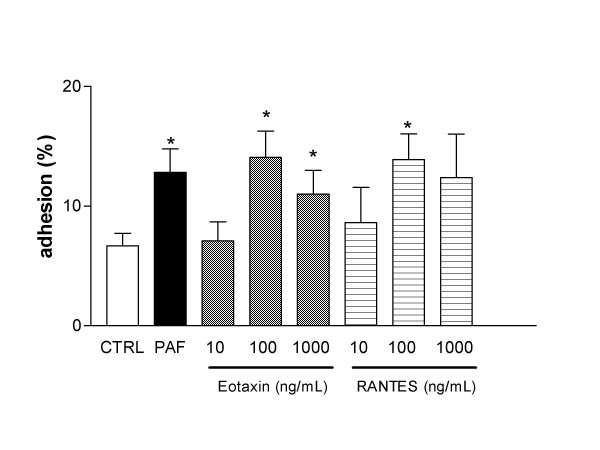
**Adhesion of human eosinophils to fibronectin-coated plates**. Eosinophils (3.5 × 10^3^cells/well) were incubated with eotaxin (10, 100 and 1000 ng/ml), RANTES (10, 100 and 1000 ng/ml) or platelet-activating factor (PAF; 10^-6 ^M) for 4 h (37°C, 5%CO_2_) and then allowed to adhere to fibronectin-coated wells for 30 min. Results are expressed as mean adhered cell percentages of total cell number ± SEM (*n *= 6). * P < 0.05 compared to control (CTRL).

Eosinophils (50 μL of 7 × 10^4 ^cells/ml) were incubated with either eotaxin (10–1000 ng/ml) or RANTES (10–1000 ng/ml) for 2, 3 and 4 h prior to addition to fibronectin-coated wells. At 2 and 3 h incubation, neither eotaxin nor RANTES significantly increased the eosinophil adhesion to fibronectin in comparison with non-stimulated cells (not shown; *n *= 6 each). On the other hand, at 4 h incubation, eotaxin and RANTES significantly (P < 0.05) increased the eosinophil adhesion compared with non-stimulated cells (Figure 1). Maximal eosinophil adhesion to eotaxin (14.1 ± 2.2%) and RANTES (13.9 ± 2.1%) was observed with the concentration of 100 ng/ml of each. In the same experimental conditions, incubation of eosinophils with PAF (10^-6 ^M), used as a positive control, significantly increased the eosinophil adhesion (12.8 ± 2.0%; Figure 1). For further experiments, the time of 4 h, and chemokine concentrations of 100 ng/ml were chosen as best incubation period and optimum concentrations.

**Figure 2:**
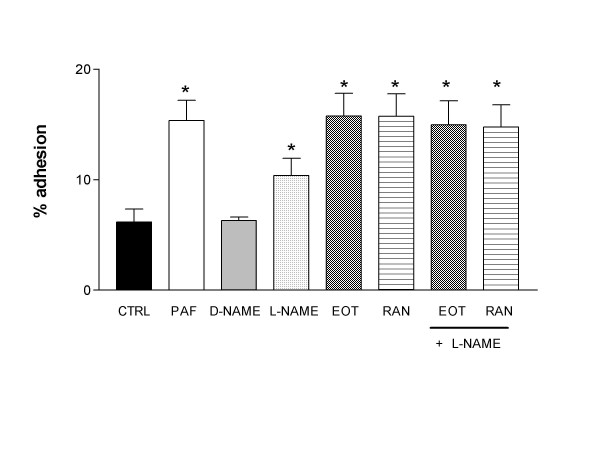
**Lack of effect of N^ω^-nitro-L-arginine methyl ester (L-NAME) upon eotaxin- and RANTES-induced eosinophil adhesion to fibronectin-coated plates**. Eosinophils (3.5 × 10^3 ^cells/well) were incubated with platelet-activating factor (PAF; 10^-6^M), D-NAME; 0.1 mM), L-NAME (0.1 mM), eotaxin (EOT; 100 ng/ml) or RANTES (RAN; 100 ng/ml) for 4 h (37°C, 5%CO_2_), and then allowed to adhere to fibronectin-coated wells for 30 min. Results are expressed as mean adhered cell percentages of total cell number ± SEM (*n *= 6). * P < 0.05 compared to control (CTRL).

Figure 2 shows that incubation of eosinophils with L-NAME alone (0.1 mM) increased significantly the adhesion of eosinophils to fibronectin (10.4 ± 1.6%; P < 0.05) compared with non-stimulated cells (6.2 ± 1.2%), whereas its inactive enantiomer D-NAME (0.1 mM) had no effect (6.3 ± 0.3%). However, co-incubation of L-NAME with eotaxin or RANTES did not affect the increased adhesion seen with the chemokines alone (Figure 2).

### Eotaxin and RANTES-induced eosinophil degranulation

Table 2 shows that incubation of human eosinophils (90 μL of 5 × 10^6 ^cells/ml) with either eotaxin (100 ng/ml) or RANTES (100 ng/ml) caused a significant cell degranulation (P < 0.001) in comparison with basal response. Incubation of eosinophils with 0.1 mM of L-NAME alone (but not with the inactive enantiomer D-NAME) also increased significantly (P < 0.001) cell degranulation when compared with non-stimulated cells. Co-incubation of L-NAME with either eotaxin or RANTES did not further affect the increased degranulation seen with the chemokines alone (Table 2). Using the same experimental conditions, the calcium ionophore A23187 (5 μM), used as a positive control, increased by 135% the EPO release above basal values.

**Table 2:** Effect of L-NAME and CC-chemokines upon eosinophil peroxidase (EPO) release.

Treatment	Optical density
CTRL	0.39 ± 0.02
A23187 (5 μM)	0.87 ± 0.04*
D-NAME (0.1 mM)	0.27 ± 0.01
L-NAME (0.1 mM)	0.70 ± 0.04*
Eotaxin (100 ng/ml)	0.70 ± 0.03*
RANTES (100 ng/ml)	0.71 ± 0.05*
Eotaxin+L-NAME	0.77 ± 0.02*
RANTES+L-NAME	0.69 ± 0.04*

Eosinophils (4.5 × 10^5 ^cells) were incubated or not (CTRL) with N^ω^-nitro-L-arginine methyl ester (L-NAME; 0.1 mM), eotaxin (100 ng/ml) or RANTES (100 ng/ml) for 4 h (37°C, 5%CO2). After incubation the released peroxidase in 50 μl of supernatant was measured and expressed as mean ± SEM of optical density (*n *= 4). *P < 0.05 compared to control (CTRL).

### Expression of integrins in human eosinophils

**Figure 3:**
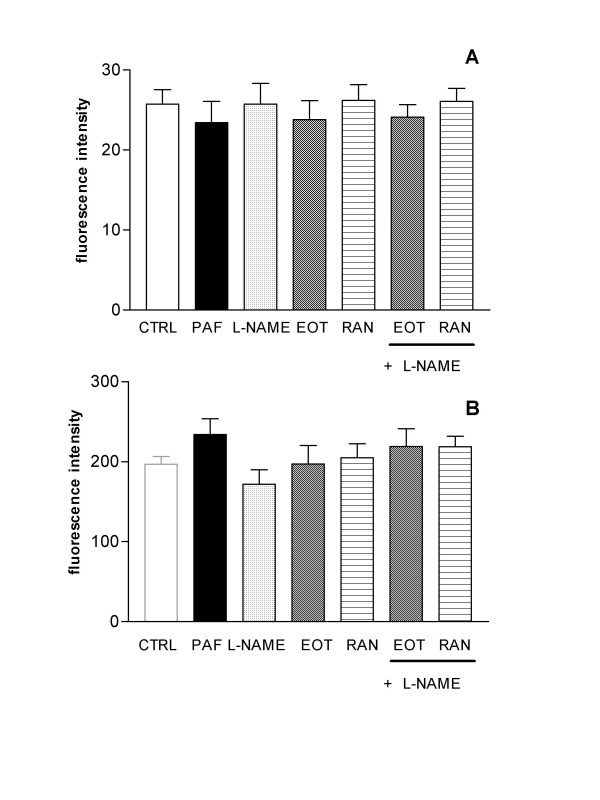
**Effect of L-NAME and CC-chemokines upon eosinophil expression of α4 and αM subunits**. Eosinophils (3.0 × 10^5 ^cells) were incubated or not (CTRL) with platelet-activating factor (PAF; 10^-6^M), N^ω^-nitro-L-arginine methyl ester (L-NAME; 0.1 mM), eotaxin (EOT; 100 ng/ml) or RANTES (RAN; 100 ng/ml) for 4 h (37°C, 5%CO_2_), and then were labelled with anti-α 4 (VLA-4; Panel A) or anti-αM (Mac-1; Panel B) antibodies. The results are expressed as mean fluorescence ± SEM (*n *= 4).

Expression of α4 (CD49d) and αM (CD11b/Mac-1) subunits on the eosinophil surface was determined by fluorescent-immunolabelling cells and detection by flow cytometry. Eosinophils, treated or not with either eotaxin or RANTES during 4 h, were incubated with the respective monoclonal antibodies, and the average mean fluorescence were detected by flow cytometry. Neither eotaxin (100 ng/ml) nor RANTES (100 ng/ml) affected the expression of α4 (Figure 3A) or αM subunits (Figure 3B) on eosinophils in any of the incubation periods.

To verify the effect of L-NAME upon integrin expression of chemokine-stimulated and non-stimulated, eosinophils were incubated for 4 h with L-NAME alone or in the presence of eotaxin (or RANTES). No changes were detected in the expression of α4 or αM subunits (Figure 3A,B).

In eosinophils treated with PAF (10^-6 ^M; used as a positive control) an increase in the αM expression about of 20% was found (fluorescence intensity of 234.1 ± 19.8) compared with untreated cells (fluorescence intensity of 196.9 ± 9.6; Figure 3B).

### Effect of chemokines and L-NAME upon intracellular cGMP levels in eosinophils

Incubation of eosinophils for either 30 min or 4 h with eotaxin (100 ng/ml) did not significantly increase the concentration of cyclic cGMP (2.9 ± 0.5 and 4.2 ± 0.5 pmol/ml, respectively) above baseline (2.3 ± 0.3 and 4.4 ± 0.5 pmol/ml, respectively; *n *= 3). Incubation of eosinophils with eotaxin (100 ng/ml each) in the presence of L-NAME (0.1 mM) also failed to affect the cGMP production (2.7 ± 0.2 and 4.3 ± 0.4 pmol/ml, for 30 min and 4 h-incubation, respectively; *n *= 3).

RANTES (100 ng/ml) also failed to significantly increase the cGMP levels above baseline (2.6 ± 0.4 and 4.5 ± 0.6 pmol/ml, respectively, for 30 min and 4 h incubation, respectively). Incubation of eosinophils with RANTES (100 ng/ml each) in the presence of L-NAME (0.1 mM) also failed to affect the cGMP production (2.1 ± 0.2 and 4.2 ± 0.4 pmol/ml, for 30 min and 4 h-incubation, respectively; *n *= 3).

Incubation of eosinophils with the NO-donor SNP (0.1 mM; positive control) markedly increased (P < 0.001) the cGMP levels above baseline at 30 min and 4 h (7.7 ± 0.7 and 6.2 ± 0.5 pmol/ml, respectively).

## Discussion

The roles of chemokines and NO in eosinophil functions in allergic processes have been largely studied, but studies focusing on the interaction between CC-chemokines (particularly eotaxin and RANTES) and the NO-cGMP pathway have been poorly explored. In the present study, using human eosinophils in vitro, our findings are clear to suggest that NO modulates neither the enhanced adhesion and degranulation in response to eotaxin or RANTES, nor the Mac-1 and VLA-4 expression in the eosinophil surface.

Among the eosinophil-active chemoattractants described to date, the CC-chemokines, particularly RANTES, eotaxin, eotaxin-2, MCP-3 and MCP-4 have been suggested to contribute to bronchial eosinophilia in patients with asthma [33,34]. In general, chemokines can induce integrin expression on the surface of leucocytes, allowing firm adhesion to the endothelium near to the inflamed focus [35], and then stimulating the transendothelial migration [36]. In murine eosinophils of IL-5 transgenic mice, eotaxin increases the CD11b and VLA-4 expressions, with maximal responses observed 1 h (CD11b) and 2 h (VLA-4) after incubation with eotaxin, suggesting that Mac-1/ICAM-1 interactions are dominant at early time, whereas VLA-4/VCAM-1 becomes predominant at later time-points during eotaxin-induced eosinophil recruitment [37]. In human eosinophils, a flow cytometry study showed that 15 min-incubation with eotaxin (300 ng/ml) caused increased surface expression of CD11b [38]. In an apparent contrast with this work, our present study showed that the CD49d and CD11b expression in human eosinophils incubated with eotaxin remained unchanged in all incubation periods studied. This discrepancy could reflect differences in the animal species (IL-5-transgenic mice vs human) and source of eosinophils used (peritoneal vs peripheral blood), as well as incubation-time used (30 min vs 4 h). On the other hand, a previous study also failed to demonstrate an increased expression of α_4 _integrin in human eosinophils treated for 30 min with eotaxin [11]. Similarly, our data from RANTES-stimulated eosinophils demonstrated no change on expression of CD49d or CD11b, which are in accordance with Jia *et al*. [37] in murine eosinophils. Additionally, in human eosinophils in vitro RANTES was shown to induce transendothelial migration without affecting the CD11b expression [17].

The action of NO is generally carried out through the activation of the soluble guanylyl cyclase, thereby increasing intracellular levels of cGMP, which acts as its second messenger. This signal transduction pathway underlies the majority of physiological actions attributed to NO, including regulation of leukocyte rolling, adhesion, and extravasation [39,40]. Immunohistochemical studies revealed that rat and human eosinophils express inducible and endothelial NO synthases, and activated rat peritoneal eosinophils are able to release NO [20,41]. Furthermore, the NO synthesis inhibitor L-NAME reduces the experimental eosinophil migration in vivo, suggesting a fundamental role for NO in eosinophil chemotactic responses [19,24] that has been associated with increased expression of MCP-1 and MIP-2 in the lung tissue [26]. On the other hand, L-NAME has been shown to enhance the in vivo cat leukocyte adhesion and the human eosinophil adhesion to fibronectin-coated plates [8,40]. However, no study exists investigating a potential role of NO-cGMP signaling pathways modulating the enhanced eosinophil adhesion in response to CC-chemokines. In the present study, at 4 h-incubation time, L-NAME itself increased the eosinophil adhesion, confirming a previous study [8]; however, L-NAME failed to affect the enhanced adhesion in response to both eotaxin and RANTES. Both of these CC-chemokines also failed to elevate the cGMP concentrations above the baseline. Additionally, treatment of eosinophils with L-NAME alone or in combination with eotaxin (or RANTES) had no effect in the cell integrin expressions (α_M _or α_4 _subunits), all suggesting that CC-chemokines eotaxin and RANTES increases the in vitro eosinophil adhesion to fibronectin-coated plates by NO-independent mechanisms. It is known that Granulocyte-macrophage colony-stimulating factor (GM-CSF) up-regulates the binding of eosinophils to VCAM-1 or fibronectin (CS-1 region) by up-regulating the binding affinity of VLA-4 to VCAM-1 (or to CS-1), which is not associated with changes in the integrin expression [11]. The binding of eosinophils to CS-1 is inhibited with neutralizing antibodies to α4 integrins expressed by eosinophils or with neutralizing antibodies to CS-1 [11]. Moreover, in the presence of eotaxin, a transient increase in the force of eosinophil adhesion to CS-1 is followed by a sustained reduction in the force of eosinophil adhesion to CS-1. This latter phase was not due to alterations in VLA-4 receptor number or alterations in VLA-4 receptor distribution [11]. This suggested to authors that conformational changes in VLA-4 receptor may be responsible for the change in binding affinity. In our study, whether such mechanism operates in L-NAME- and CC-chemokines-treated human eosinophils requires additional studies. We also would like to emphasize that a small fraction of stimulated cells were able to adhere, which seems to be a common feature of in vitro models exploring cell adhesion. The basal (non-stimulated) adhesion in models of serum-, fibrinogen-, fibronectin- or recombinant VCAM1-coated wells ranges always from 5 to 30%, depending of the cell type used (eosinophils, neutrophils, platelets) and experimental conditions [8,42-46]. Most importantly, cell stimulation with different agents usually enhances the adhesion about of 20–100% above baseline.

Eotaxin and RANTES are also important chemotactic factors for eosinophils, and are reported to largely contribute to the movement of eosinophils from the blood to the sites of inflammation. Once recruited to tissues, eosinophils receive signals to promote degranulation, causing the release of eosinophil granule proteins such as major basic protein (MBP), eosinophil peroxidase (EPO) and eosinophil cationic protein (ECP) that has been associated with the induction of epithelial damage, edema, and airways hyperreactivity, playing therefore a pivotal role in the pathogenesis of allergic disorders [47]. Accordingly, the increased presence of cationic granule in the asthmatic lung correlates with severity of bronchial asthma [48,49]. Amongst the inflammatory mediators postulated to contribute to eosinophil degranulation, eotaxin and/or RANTES have been shown to release CCR3-dependent granule proteins [50,51] in equivalent amounts to PAF, which is considered a potent eosinophil activator [52]. In a human eosinophil cell line (15-HL-60), eotaxin, eotaxin-2 and eotaxin-3 also produced concentration-dependent degranulation of EPO [53]. Since the chemokines may reach high amounts in the allergic inflammatory site, it is accepted that they greatly contribute to local eosinophil degranulation [52]. Our findings showed that both eotaxin and RANTES caused a significant eosinophil degranulation, thus confirming the literature data. However, the failure of L-NAME to modify the eotaxin- and RANTES-induced degranulation indicates that NO does not modulate degranulation in CC-chemokine-activated eosinophils. It is interestingly, however, that L-NAME itself significantly increased the EPO release in equivalent amounts to the CC-chemokines. While in other cell types such as mast cells, NO synthesis inhibitors are reported to cause cell degranulation [54,55], to our understanding the present study is the first to show that in vitro NO inhibition in human eosinophils leads to cell degranulation. It is known that exocytosis of stored granule proteins of eosinophils takes place by three distinct mechanisms, namely classical exocytosis, piecemeal degranulation and cytolysis [56]. Further studies are thus required to elucidate the mechanism(s) by which NO influences the eosinophil degranulation.

## Conclusion

In summary, our study shows that the CC-chemokines eotaxin and RANTES increases the in vitro eosinophil adhesion and degranulation by NO-independent mechanisms. The failure of CC-chemokine to affect VLA-4 and Mac-1 expression on eosinophil surface indicates that changes in the integrin function (avidity or affinity) are rather involved in the enhanced adhesion.

## Competing interests

The authors declare that they have no competing interests.

## Authors' contributions

LL carried out the functional studies and helped to draft the manuscript. GF and AN participated in the flow cytometry. CA–N, AZ and GdN participated in the design of the study. EA participated in its design and supervision, and helped to draft the manuscript. All authors read and approved the final manuscript.

## Pre-publication history

The pre-publication history for this paper can be accessed here:


